# Suppressed without a Cause: A Case of Idiopathic Immune Deficiency

**DOI:** 10.7759/cureus.2009

**Published:** 2018-01-01

**Authors:** Muhammad Talha Ayub, Munnam S Jafar, Muhammad Khalid, Muhammad A Baig, Benjamin Mba

**Affiliations:** 1 Internal Medicine, John H Stroger J. Hospital of Cook County; 2 Internal Medicine, Jinnah Hospital Lahore (JHL)/Allama Iqbal Medical College (AIMC), Lahore, Pakistan.; 3 Internal Medicine, East Tennessee State University

**Keywords:** idiopathic, lymphopenia, meningitis

## Abstract

We report a case of a 45-year-old male who presented with a headache, fever, vomiting, somnolence, and difficulty walking for 10 days. His cerebrospinal fluid studies revealed cryptococcal meningitis. Chest and abdominal computed tomography (CT) scans showed splenomegaly along with mediastinal, retroperitoneal and inguinal lymphadenopathy. CD4 count turned out to be 208 μL^−1^. Human immunodeficiency virus (HIV) testing, serum protein electrophoresis, serum light chains and quantitative immunoglobulins were non-diagnostic and CD4 lymphopenia was attributed to acute infection. However, a persistent CD4 lymphopenia was seen in subsequent outpatient testing, which prompted a detailed workup for secondary causes of immunodeficiency. Repeated lymph node biopsies with analytic cytometric immunophenotypic analysis were normal, as was the bone marrow biopsy with detailed immunophenotypic and cytogenetic studies. The patient was hence being treated as a case of idiopathic CD4 lymphocytopenia.

## Introduction

Immunodeficiency is an inability to mount a normal immune response against a specific antigen in the form of antibodies or sensitized T cells. It can be congenital or acquired. Idiopathic CD4 lymphocytopenia (ICL) is a condition defined as persistent CD4 lymphocyte depletion (absolute CD4 count <300 μL^−1^ or <20% of total lymphocytes on two separate occasions 1-3 months apart) in the absence of human immunodeficiency virus (HIV) or any defined immune-deficiency disease or therapy [1].

## Case presentation

A 45-year-old male with no past medical history presented with an occipital headache for 10 days. It was associated with fever, chills, nausea, vomiting, slurred speech, somnolence and difficulty walking. He denied focal weakness, paresthesia, neck stiffness, visual changes, chest pain, shortness of breath, urinary and bowel incontinence. The patient was in a monogamous heterosexual relationship. He endorsed a history of social drinking but denied smoking or drug abuse. Medication history was unremarkable.

**Figure 1:**
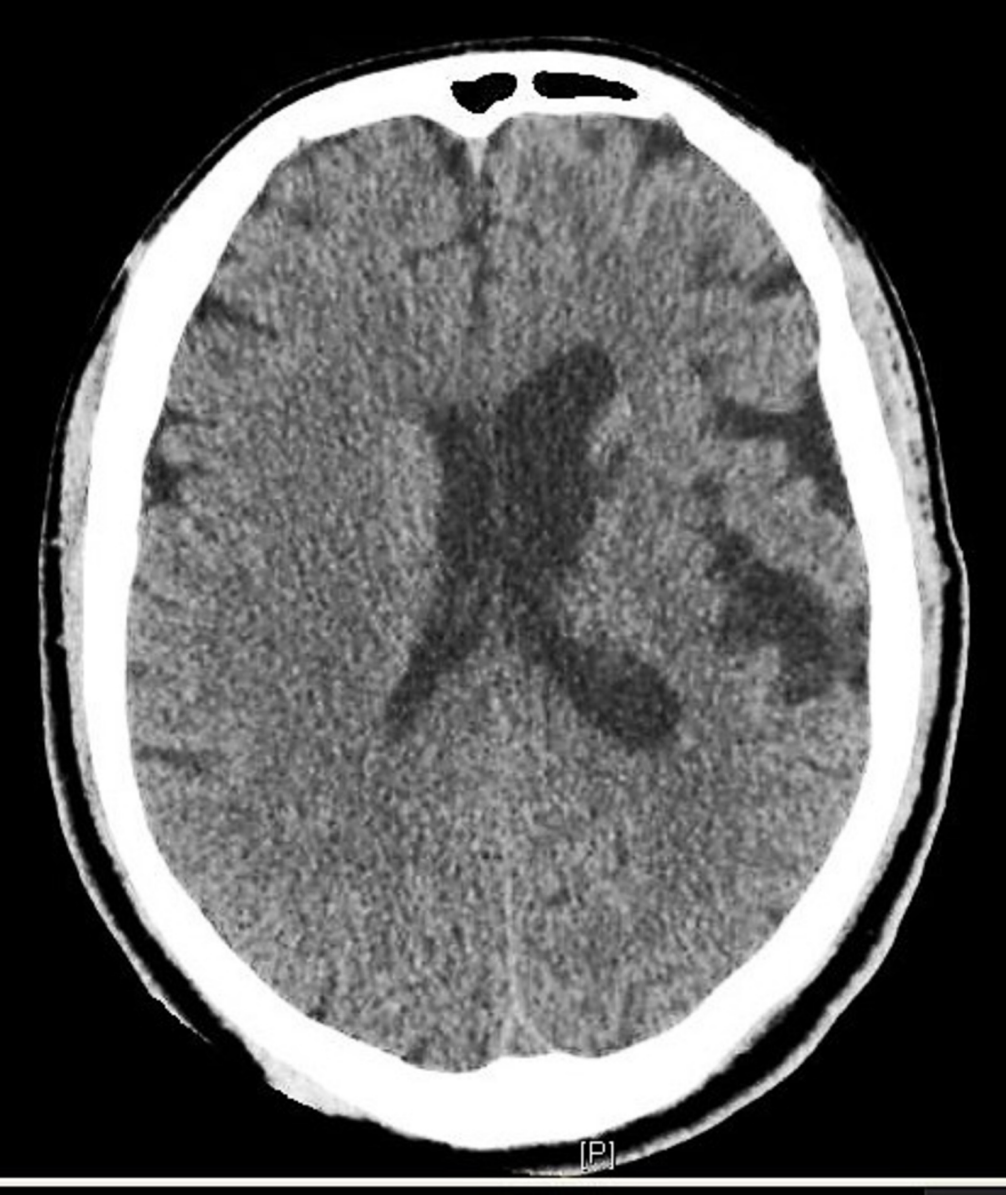
Computed tomography (CT) scan head without contrast showing ex vacuo dilatation of left lateral ventricle.

The patient was afebrile with a heart rate of 88 bpm, RR 21/min, and BP 136/97 mmHg. Physical exam was unremarkable except for pallor and inguinal lymphadenopathy. Labs were significant for hypotonic hyponatremia and normal complete blood count (CBC). Computed tomography (CT) scan head without contrast showed ex vacuo dilatation of the left lateral ventricle (Figure 1).

Lumbar puncture was done and cerebrospinal fluid (CSF) analysis unveiled cryptococcal meningitis. Multifocal nodularities involving bilateral basal ganglia consistent with cryptococcomas, were present on magnetic resonance imaging (MRI), along with leptomeningeal enhancement and chronic lacunar infarcts. Induction chemotherapy was started with amphotericin and flucytosine.

CT chest, abdomen and pelvis reported diffuse mediastinal, retroperitoneal, inguinal lymphadenopathy with splenomegaly. Immunodeficiency workup revealed a CD4 count of 208 μL^−1^. HIV testing, serum protein electrophoresis, serum light chains and quantitative immunoglobulins were non-diagnostic. CD4 lymphopenia was attributed to acute infection and the patient was sent to a nursing home for two weeks of IV amphotericin therapy. However, he had persistently low absolute CD4 count ranging between 161 and 213 μL^−1^ on outpatient follow-up.

Three serial lymph node biopsies for etiological workup of CD4 lymphopenia were nonconclusive, revealing a normal analytic cytometric immunophenotypic analysis. A bone marrow biopsy with immunophenotypic and cytogenetic studies (including karyotype, BCR/ABL1, JAK2 V617F, exon 12 mutations, fluorescent in situ hybridization (FISH) for platelet-derived growth factor receptor (PDGRF) alpha, beta, and fibroblast growth factor receptor 1 (FGFR1)) was normal. IgH gene rearrangement studies showed a weak peak on B cell framework III on repeat assays, not strong enough to be termed as monoclonal. T cell receptor (TCR) gamma gene arrangement did not point towards a definite diagnosis. Moreover, a review of the sample by the National Institutes of Health (NIH) also proved to be inconclusive. The patient was hence labeled as a case of idiopathic CD4 lymphocytopenia.

After management of acute meningitis, he was discharged on fluconazole and augmentin therapy. The patient had a stable CD4 count without features of any immunodeficiency-related illness on the subsequent outpatient visits but was lost to follow up after three months of initial presentation. Attempts to contact the patient were unsuccessful, however, emergency contact (friend) revealed that he returned to India and was hospitalized for rapidly progressive dyspnea and confusion. He later died of multiorgan failure after 15 days of MICU stay.

## Discussion

ICL was first defined by the Center for Disease Control and Prevention (CDC) in 1992 as low CD4 cell count in the absence of HIV and other secondary causes of immunodeficiency. Idiopathic CD4 lymphopenia is a rare immunological disorder associated with the persistent low CD4 count. The mean age at diagnosis is 41 year with slight male predominance [1]. Diagnostic criteria for ICL include a CD4 count < 300 cells or <20% of the total lymphocyte count, in the absence of HIV infection and secondary evidence of immunodeficiency conditions associated with low CD4 count [1,2]. In contrast to HIV, there is a slow progressive decline in the CD4 count. The pathogenesis is not well defined, but proposed mechanisms include: defective T cell production and differentiation, early maturation arrest, increased apoptosis, defective production of interferon gamma and increase destruction of cells due to sequestration in spleen and lymph node.

The diagnosis of ICL should be sought when an opportunistic infection is diagnosed in an otherwise healthy individual. ICL predominantly presents with opportunistic infections like cryptococcus, human papillomavirus (HPV), and non-tuberculous mycobacterial species, especially Mycobacterium avium complex (MAC) [2-5]. Patients are also more prone to Epstein-Barr virus (EBV) related B-cell and central nervous system lymphomas, primary pleural effusion and Kaposi’s sarcoma. Loss of immune tolerance increases vulnerability to autoimmune diseases like antiphospholipid antibody syndrome, autoimmune hemolytic anemia, ulcerative colitis, Grave's disease, vitiligo, autoimmune thyroiditis, Behçet's disease, and vasculitis [3]. In addition to the aforementioned, skin disorders such as psoriasis and central nervous system conditions like progressive multifocal leukoencephalopathy have also been reported in association with ICL [6,7]. ICL is a diagnosis of exclusion so secondary causes of lymphopenia must be excluded to arrive at the diagnosis. The most common secondary causes are viral and bacterial infections, chemotherapy and cytotoxic drugs, malignancy and autoimmune conditions (Table 1). Our patient had immunodeficiency defined illness, cryptococcal meningitis, in the absence of any secondary causes of immune suppression as evident by extensive workup.

**Table 1:** Conditions associated with low CD4 count.

Conditions associated with low CD4 levels
Autoimmune	Sjögren disease	Systemic lupus erythematosus	Kikuchi disease
Drugs	Corticosteroids	Chemotherapy	Cytotoxic immunosuppressants
Genetic syndromes	XMEN (X-linked magnesium deficiency with Epstein-Barr virus infection and neoplasia)	WHIM (warts, hypogammaglobulinemia, infections, and myelokathexis)	Unc119 mutation (uncoordinated 119)
Hematologic conditions	Lymphoma	Myelodysplastic syndromes	Aplastic anemia
Infections	HIV-1 and HIV-2 (human immunodeficiency virus)	Human T-cell lymphotropic viruses 1 and 2	Mycobacterium tuberculosis	Epstein-Barr virus	Cytomegalovirus	Human herpes virus 6	Recalcitrant warts
Various	Common variable immune deficiency	Sarcoidosis	Radiation therapy

There is no consensus for diagnostic investigations of ICL but the workup should be dictated by the presenting opportunistic infection and generally should include CD4, CD8, natural killer, and B-cell subsets, immunoglobulin levels, HIV 1-2 serology and polymerase chain reaction (PCR), human T-lymphotropic viruses type I (HTLV-I) and type II (HTLV-II) testing, EBV PCR, cytomegalovirus (CMV) PCR, and an autoantibody panel. The diagnosis is especially challenging in the presence of autoimmune diseases, since lymphopenia can be secondary to autoimmunity and vice versa, with transient immunosuppressant therapy-induced lymphopenia further complicating matters [4].

Although no published guidelines for the management of ICL exist, it is logical to assume that primary and secondary prophylaxis and treatment of opportunistic infections should form the mainstay of therapy. Our patient was discharged on antifungal and gram-positive therapy and was in remission before he was lost to follow up. Prophylaxis strategies generally follow recommendations for HIV. Live vaccines are contraindicated and should be avoided. Interleukin 2, gamma interferon and hematopoietic cell transplantation have been successfully used to augment the CD4 count in ICL whereas interleukin 7 is currently being studied [3]. Interleukin 2 and gamma interferon-mediated increase in CD4 counts improved clinical response to opportunistic infections such as cryptococcal meningitis and non-tuberculous mycobacterial infections. A study done in December 2015 with interleukin 7 showed promising results [8,9].

In contrast to HIV, ICL patients have a good prognosis and the progressive decline in CD4 counts is slow. Based on prospective studies, close monitoring with CD4 counts every four months is suggested in stable patients, especially for the first three years of diagnosis as there is a risk of serious infections and a possibility of normalization of CD4 T-cell counts within three years [6]. Our patient had a poor prognosis and died within six months of diagnosis from multiorgan failure, emphasizing the importance of close follow-up for early detection of life-threatening infections. Appropriate screening tests for chronic HPV infection-related squamous cell carcinoma should be employed as well. Low CD8 count (<180/mm^3^) and the degree of CD4 T-cell activation (measured by HLA-DR expression) are associated with adverse outcome (opportunistic infection-related death) [3].

## Conclusions

ICL is a rare idiopathic condition which should be suspected when an opportunistic infection is detected in an otherwise healthy individual, which was cryptococcal meningitis in our case. Patients are more prone to develop malignancies and autoimmune diseases. A thorough workup excluding immunologic, hematologic, rheumatologic, and infectious etiologies should be carried out to establish a diagnosis of ICL. Our patient had extensive workup to reach this diagnosis of exclusion. Prophylaxis and treatment of opportunistic infections, regular follow-up with CD4 count and augmentation of immunity with interleukin 2, hematopoietic cell transplantation, interferon gamma and interleukin 7 therapy form the mainstay of management.
